# Supplementary material for: Neural activity induces strongly coupled electro-chemo-mechanical interactions and fluid flow in astrocyte networks and extracellular space—A computational study
Source: PLoS Comput Biol. 2023 Jul 21;19(7):e1010996. doi: 10.1371/journal.pcbi.1010996 (PMC10396022; doi:10.1371/journal.pcbi.1010996)
Supplement: Attachment — Submitted filename: Response to reviewers.pdf [file pcbi.1010996.s001.pdf]

Dear Editor,

We hereby resubmit our manuscript “Neural activity induces strongly coupled electro-chemo-mechanical interactions and fluid flow in astrocyte networks and extracellular space - a computational study” by Marte J. Sætra, Ada J. Ellingsrud, and Marie E. Rognes for publication in PLoS Computational Biology.

We thank the reviewers for their feedback and constructive comments. Our detailed response follows below. The reviewers’ comments are included in blue, while our responses follow in black text. Also attached is a version of the manuscript indicating the changes made from the previous version.

Reviewer #1: This manuscript presents a numerical study of fluid flow, ion motion, and associated pressures and electrical potentials, in and around an astrocyte in the brain. […] The modeling and manuscript are thorough and meticulous. The results are clear enough and extend significantly beyond prior studies, which have not accounted for coupling among so many mechanisms. The interpretations are straightforward and not overwrought. Thus I strongly support publication, providing the authors respond effectively to a few questions and comments:

Comment: We thank the reviewer for this prompt and positive evaluation of our manuscript.

Reviewer #1: Figure 2 shows temporal variations that look like smooth exponential rise and decay in every case except one, the concentration of K+ ions (panel D). What is happening there?

Comment: We thank the reviewer for this observant question. The behavior of the intracellular potassium concentration is a direct effect of the volume dynamics. When we turn the input currents on, potassium flows into the cell (via the pump), as shown in panel A below. The inward potassium flux leads to a steady rise of intracellular potassium, as we see if we plot $[K^+]$*alpha, that is, the amount of potassium per total volume (panel B below). However, the potassium concentration shown in Figure 2D (amount of potassium per intracellular volume) is not only a function of the potassium currents (intracellular and transmembrane) but also of the intracellular volume fraction (panel C below). For this reason, $[K^+]$ may decrease, although $[K^+]$*alpha increases. By dividing $[K^+]$*alpha (panel B) by alpha (panel C), we get the observed dynamics (panel D). To exclude the possibility of numerical artifacts, we made the same simulation/calculations using N = 800 and dt = 1e-4 and it did not make any difference.

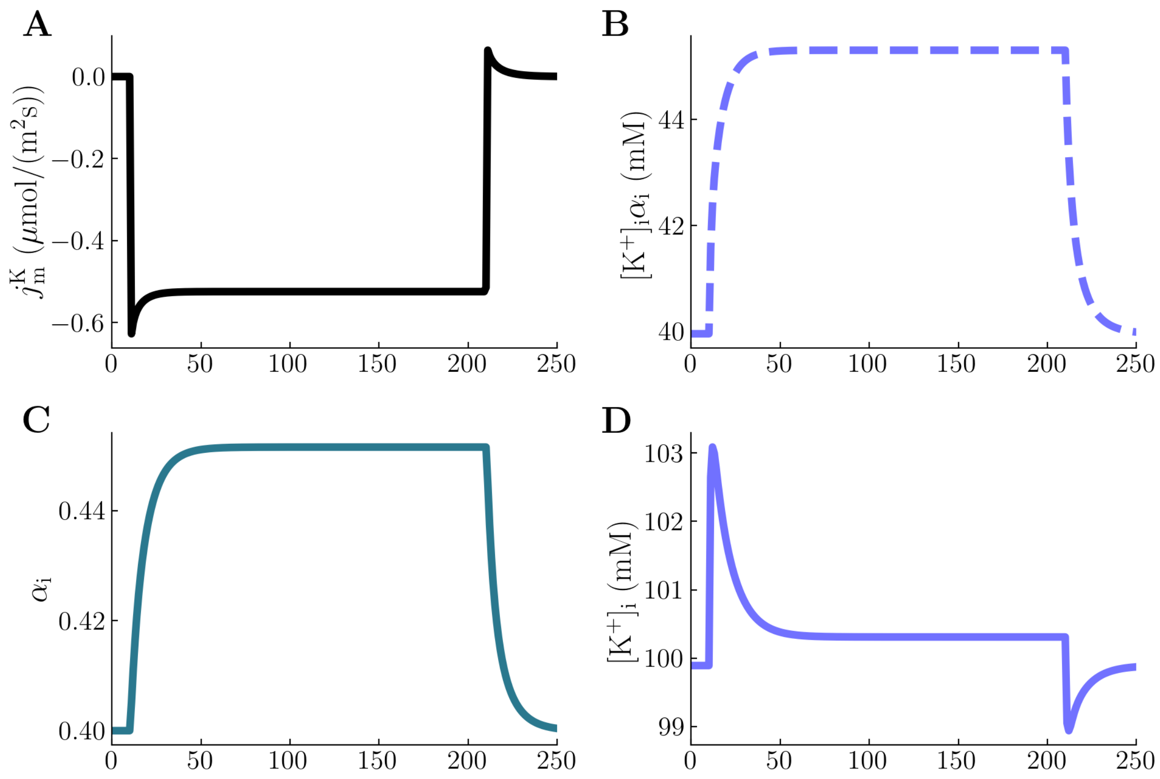

Reviewer #1: The results consistently show that osmotic pressures are much higher than hydrostatic pressures. How much error would be introduced if hydrostatic pressures were neglected altogether?

Comment: The reviewer is right that the hydrostatic pressures across the membrane are consistently smaller than the osmotic pressures. However, the intra- and extracellular gradients are on the same order of magnitude as the other forces and a prerequisite for intra-compartmental fluid flow as the hydrostatic pressures (in this model) are set up to ensure that the superficial fluid velocities are divergence-free (Equation 7). To neglect the hydrostatic pressures altogether then becomes a question about the role of the intra-compartmental fluid flow. We discuss this question throughout the paper, both by comparing the flow models M1, M2, and M3 to the zero-flow model M0, but also by comparing the different flow models (that is, models that predict different strengths of the fluid velocities).

Reviewer #1: The scenarios simulated all consider quasi-steady behaviors between step changes in neuronal activity (turning on at 10 s, then turning off at 210 s). Of course, many other timescales are of interest neurologically. Most of all I wonder what the model would predict for slow / ultraslow frequencies of the sort considered by Fultz et al. (Ref. 1). Could the authors run the model with neuronal activity varying at 0.05 Hz or 1 Hz? Or at least speculate in the Discussion about what might occur?

Comment: This is a very interesting point brought up by the reviewer. Large-scale neural wave patterns may not directly translate to local ionic waves, but studying pulsatile stimuli is an intriguing scenario that we chose to investigate further.

Action: We have run two new simulations with the same stimulus zone and same stimulus amplitude as before, but now with sinusoidal waves of K^+ input fluxes, with frequencies of 1 Hz and 0.05 Hz. We found that slow and ultraslow stimuli induce pulsatile changes in the observed dynamics, including pulsatile fluid flow. The slow and ultraslow stimuli express different characteristics in wave amplitudes and phase shifts. The findings are described in a new section titled 2.8 Fluid flow patterns induced by low-frequency activity waves (page 9). The new stimulus protocols are added to Section 4.5 in Methods (page 19), and we have extended the Discussion (page 13) with a paragraph discussing the results in relation to the literature, including but not restricted to Fultz et al. (2019).

Reviewer #1: Finally I noticed (just two) typos:
line 387 - acknowledges should be acknowledge
line 153 - stabilize should be stabilizing

Comment: We thank the reviewer for pointing this out. This is now fixed.

Reviewer #2: Interest in the nature and drivers of fluid flow within the brain has taken on renewed interest with the idea that this movement may be important for clearance of waste from the brain through the glymphatic system. Because these flows are technically difficult to measure, they have been the focus of many computational models, though most of them have focused on mechanical drivers (pulsation, etc.). This paper investigates the intra and extracellular fluid flows induced by ionic/osmotic currents (driven by local neural activity) in simple model of the astrocyte syncytia and extracellular space. The authors find that there is fluid flow away from the site of activity and into the astrocytes remote from the site. The authors find that including osmotic and electrodiffusion in their models have a large effect on flow. There has been very little work done in this area and this looks to be an important contribution.

Comment: We thank the reviewer for this prompt and constructive evaluation of our manuscript.

Reviewer #2: The 6.7mM K+ external change that is used here is very high, well beyond the standard physiological range. Increases in vivo are more in the range of 1 mM (see (Rasmussen, 2019) for some measurements on naturalistic conditions). That being said, looking at the equations I do not see anything that would prevent the flows scaling linearly (or something like that) with linear increases in injected /withdrawn K+/Na+. Can the authors address this point, either analytically or with simulations if this is the case?

Comment: We thank the reviewer for the opportunity to clarify. The input fluxes are set such that the extracellular potassium concentration reaches a maximum value of approximately 10 mM. The reasoning behind this choice is that we want the potassium buffering process to play a significant role while at the same time stay below the more extreme concentration levels that have been observed in conditions such as spreading depression.

Action: The 6.7 mM change in extracellular potassium concentration is already justified in Section 4.5, but to make it easier for the reader to be aware of our reasoning, we have moved the paragraph in question to Section 2.1 (page 5). In addition, we have investigated the relationship between the input flux density and the superficial fluid velocities by running new simulations with 20%, 40%, 60%, 80%, and 100% of the default stimulus strength. We find a linear relationship between the potassium input flux density and the superficial fluid velocities and have extended Section 2.9 (Flow sensitivity to changes in permeabilities, stiffness, and input flux density) with these findings (page 11). We refer to the manuscript for the detailed changes.

Reviewer #2: Will multiple areas of activity lead to a linear superposition of the observed dynamics?

Comment: This is an intriguing question from the reviewer, which prompted us to study this further. Specifically, we ran three new simulations using modeling setup M3, with the only difference being the position of the input zone(s). The setups of the input zones were as follows:

S1: one input zone, centered in the *left* part of the domain
S2: one input zone, centered in the *right* part of the domain
S3: two input zones, corresponding to S1 + S2.

To assess whether multiple areas of activity lead to a linear superposition of the observed dynamics, we plotted selected predictions from S3 together with predictions from S1 and S2 added together. The figure below displays spatial profiles of the K^+ input flux (A), the K^+ decay flux (B), changes in the extracellular- (C) and intracellular (D) ion concentrations, changes in the extracellular (E) and intracellular (F) volume fractions, change in the transmembrane hydrostatic pressure (G), and change in the membrane potential (H) at t=200s for S3 (solid lines) and S1+S2 (dashed lines). We observe that the solid and dashed lines overlap or almost overlap for the extracellular ion concentrations (Na^+, K^+, and Cl^-), intracellular Cl^- concentration, volume fractions, and pressure. In contrast, the solid and dashed lines deviate from each other for the intracellular Na^+ and K^+ concentrations and the membrane potential. Due to the nonlinearity of the system, we assume that the precise relationship between S3 and S1+S2 will depend on the specific simulation setup (i.e., stimulus strength, etc.).

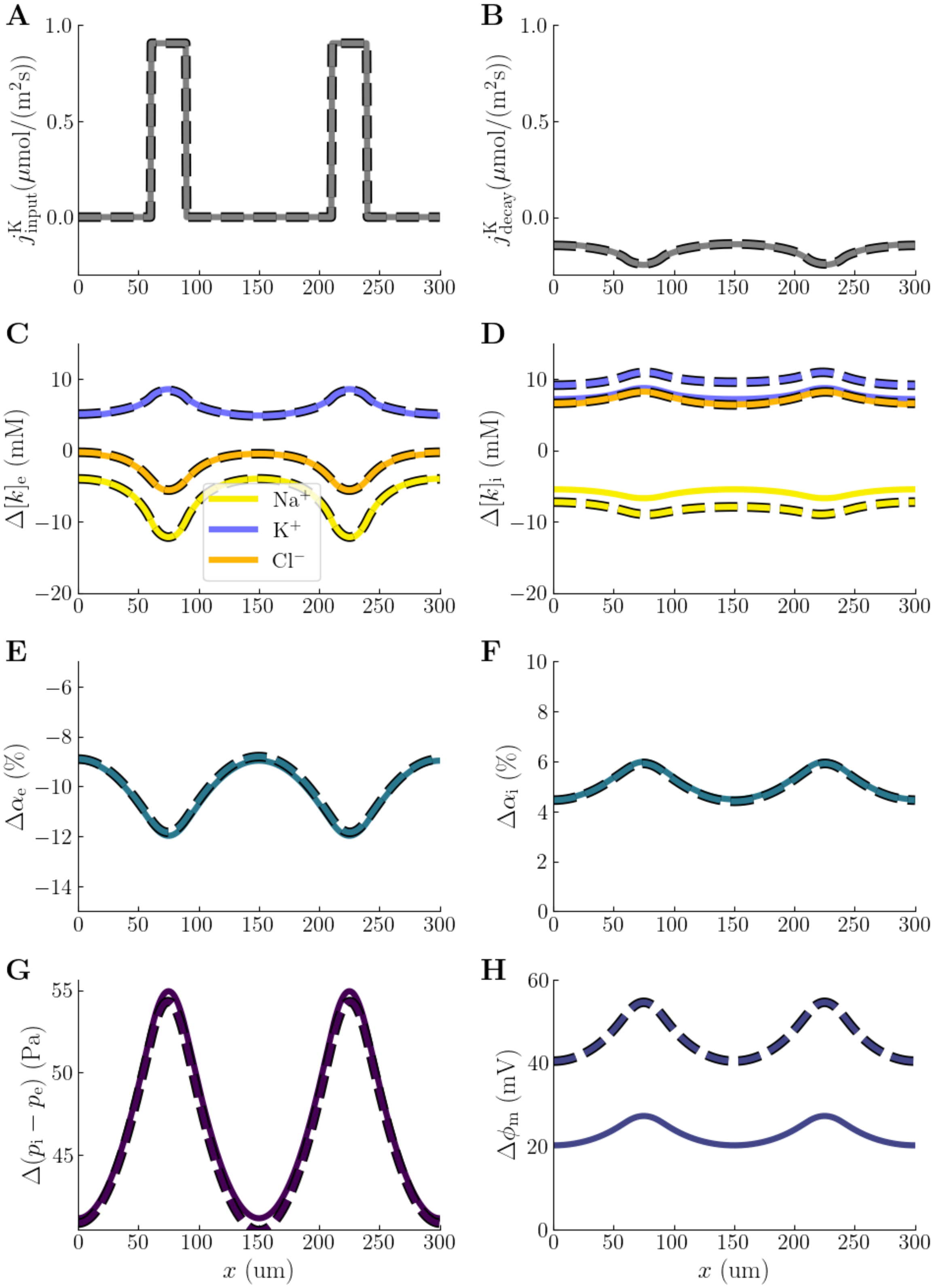

Reviewer #2: The abstract and summary are vague and do not contain concise descriptions of the results. They should be re-written so that what is shown in the paper is summarized in the abstract.

Comment/Action: We note the reviewer's comments. To make the descriptions more concise, we have added the following summary to the abstract and a similar summary to the end of Section 1 (page 3):

"We find that neuronal activity alone may induce intracellular fluid velocities in astrocyte networks of up to 14 µm/min, and fluid velocities in the ECS of similar magnitude. These velocities are dominated by an osmotic contribution in the intracellular compartment; without it, the estimated fluid velocities drop by a factor of ×34–45. Further, the compartmental fluid flow has a pronounced effect on transport: advection accelerates ionic transport within astrocytic networks by a factor of ×1–5 compared to diffusion alone."

Reviewer #2: I think the paper would be strengthened by exploring a different set of boundary conditions. The no-flow conditions here are fine as a simplification, but in the real system one might expect a constant pressure/ionic concentration boundary that might produce different responses, particularly if the activity location is moving. The "holy grail" of all this glymphatic system modeling is to get directed fluid flow (left to right or vice versa in this model's extracellular space). Is there any way the authors could generate something like this, or suggest experiments that could test their ideas?

Comment: As the reviewer mentions, in this study, we consider no-flow boundary conditions. We absolutely agree that this is clearly a simplification and that other boundary conditions would be interesting to explore. Indeed, we are particularly interested in exploring the interplay between pressure pulsations at different frequencies and amplitudes, electrochemical activity, and fluid flow – within the tissue or across the boundaries. The domain boundaries could then represent e.g the vasculature or perivascular spaces bounded by astrocyte endfeet. However, there are (at least) two important methodological aspects that must be resolved before addressing this context successfully. First, the pressures induced by concentration differences/osmotics are substantial in comparison with the hydrostatic pressure differences associated with cardiovascular pulsations; the magnitude of these forces must be calibrated and reconciled. Second, the model (as the system itself) describes a nonlinear interplay between chemical, electrical and mechanical effects which easily may display instabilities, in particular in connection with boundary conditions admitting substantial fluid influx/efflux. Care should therefore be taken when choosing appropriate boundary permeabilities and counter pressures. We very much plan to explore this in more detail in follow-up studies, and therefore consider this to be outside the scope of the current manuscript.

Action: We have extended the Discussion with a sentence relating to the choice of boundary conditions (page 14).

Reviewer #2: Don't use dotted lines- it makes it hard to see sharp transitions.

Comment/Action: Fixed.

Reviewer #2: A zoom in on the onset offset dynamics in figure 2 would be helpful for the reader to understand the timescale of the changes here.

Comment/Action: Fixed

Reviewer #2: Mark default values on figure 7.

Comment/Action: Fixed.
